# Language choice in bimodal bilingual development

**DOI:** 10.3389/fpsyg.2014.01163

**Published:** 2014-10-20

**Authors:** Diane Lillo-Martin, Ronice M. de Quadros, Deborah Chen Pichler, Zoe Fieldsteel

**Affiliations:** ^1^Department of Linguistics, University of ConnecticutStorrs, CT, USA; ^2^Haskins LaboratoriesNew Haven, CT, USA; ^3^Departamento de Libras, Universidade Federal de Santa CatarinaFlorianópolis, Brazil; ^4^Department of Linguistics, Gallaudet UniversityWashington, DC, USA; ^5^Department of Linguistics, Brown UniversityProvidence, RI, USA

**Keywords:** bimodal bilingualism, bilingual development, code-blending, language mixing, interlocutor sensitivity

## Abstract

Bilingual children develop sensitivity to the language used by their interlocutors at an early age, reflected in differential use of each language by the child depending on their interlocutor. Factors such as discourse context and relative language dominance in the community may mediate the degree of language differentiation in preschool age children. Bimodal bilingual children, acquiring both a sign language and a spoken language, have an even more complex situation. Their Deaf parents vary considerably in access to the spoken language. Furthermore, in addition to code-mixing and code-switching, they use code-blending—expressions in both speech and sign simultaneously—an option uniquely available to bimodal bilinguals. Code-blending is analogous to code-switching sociolinguistically, but is also a way to communicate without suppressing one language. For adult bimodal bilinguals, complete suppression of the non-selected language is cognitively demanding. We expect that bimodal bilingual children also find suppression difficult, and use blending rather than suppression in some contexts. We also expect relative community language dominance to be a factor in children's language choices. This study analyzes longitudinal spontaneous production data from four bimodal bilingual children and their Deaf and hearing interlocutors. Even at the earliest observations, the children produced more signed utterances with Deaf interlocutors and more speech with hearing interlocutors. However, while three of the four children produced >75% speech alone in speech target sessions, they produced <25% sign alone in sign target sessions. All four produced bimodal utterances in both, but more frequently in the sign sessions, potentially because they find suppression of the dominant language more difficult. Our results indicate that these children are sensitive to the language used by their interlocutors, while showing considerable influence from the dominant community language.

## Introduction

There has been much interest in how the languages of children developing as simultaneous bilinguals separate and interact. It has frequently been observed that, especially at the earliest ages, children may seem to mix their languages, by using structures that apparently combine elements of both (Grosjean, 1989; Bhatia and Ritchie, 1999; Paradis, 2001). In addition, children may interact with speakers of one of their languages (say, language A) using elements of their other language (say, language α)—showing incomplete discourse separation (Paradis and Nicoladis, 2007). Such observations have led to the proposal that bilingual children's language is “fused” at an early age (Volterra and Taeschner, 1978); that is, they have one grammar with elements of both languages.

However, many authors have argued against the view that bilingual children's languages are “fused” (Genesee, 1989). They observe, for example, that even highly fluent bilingual adults produce “mixed” structures showing elements of both languages. Adult bilinguals who are fully proficient in both languages allow the languages to interact in varied and interesting ways (Costa and Santesteban, 2004; Bishop and Hicks, 2005; Gonzalez-Vilabazo and López, 2012). Code-switching is taken as a sign of bilingual proficiency (Poplack, 1980; Lucas and Valli, 1992), and it is heavily used as an in-group sociolinguistic phenomenon in highly bilingual communities (Bhatt and Bolonyai, 2011). Nevertheless, it cannot be said that young bilingual children's languages are completely separate (Unsworth, 2013). We conclude, then, that the best tack to take toward understanding the development of bilingualism is to model the adult state and to see how children move toward achieving this state.

Our project takes this approach: we are developing a model of bilingualism that we expect applies equally to describing both adult and child states, although some of the details of grammatical knowledge for children may be different from that of adults. Our project also takes one further step: while it should also apply to unimodal bilingualism, we are developing this model in the context of bimodal bilingualism: children who are becoming bilingual in a signed language and a spoken language (for a general overview on such children, see Baker and van den Bogaerde, 2014). Bimodal bilinguals can be hearing (using the spoken and written form of a spoken language) or Deaf (some using both forms, others using only the written form of a spoken language). They include people who use a sign language casually, daily, or professionally as interpreters. Most of the children we are studying—and all of the ones in the current report—have normal hearing, but their families (in particular, one or both parents) are Deaf and use a sign language with them. The children acquire sign language at home, and they acquire spoken language from the greater community (including other relatives, neighbors, schools, etc.). Then, we ask how the issues around language separation and mixing are different in the context of bimodal bilingualism.

The few existing studies with adult bimodal bilinguals have led to several conclusions. First, as with unimodal bilinguals, both of the languages of bimodal bilinguals are active and influence language use and processing, even in contexts that only call for one language (Kroll and Stewart, 1994; Kroll et al., 2006; Emmorey et al., 2008b; Shook and Marian, 2012). The various types of language mixing observed in unimodal bilinguals can also be found, but with a twist. Code-switching—in this context, ceasing production in one language (e.g., speech) and starting up in the other language (e.g., sign)—is relatively rare. Emmorey et al. (2008a) studied adult bimodal bilinguals, often known as codas (“Child of Deaf adult,” implied hearing and adult), in a highly bilingual context (conversing with another familiar coda). Overall, their participants produced code-switches in only 6.26% of the utterances analyzed. However, they displayed another type of language “mixing,” unique to bimodal bilinguals: code-blending. Code-blending is the natural and spontaneous use of speech and sign together. In the data collected by Emmorey et al. (2008a) 35.71% of all utterances contained code-blending. Finally, Emmorey et al. also observed the use of sign language structures in the spoken language—so-called cross-linguistic influence, or transfer—another type of language “mixing.”

Bimodal bilinguals introduce a new type of “mixing” to the picture of how the languages of a bilingual interact. Not only do they produce structures showing cross-linguistic influence and code-switching, they also productively use code-blending. Any model of bilingualism—the target toward which children develop—must account for all three of these phenomena.

**Figure 1:**
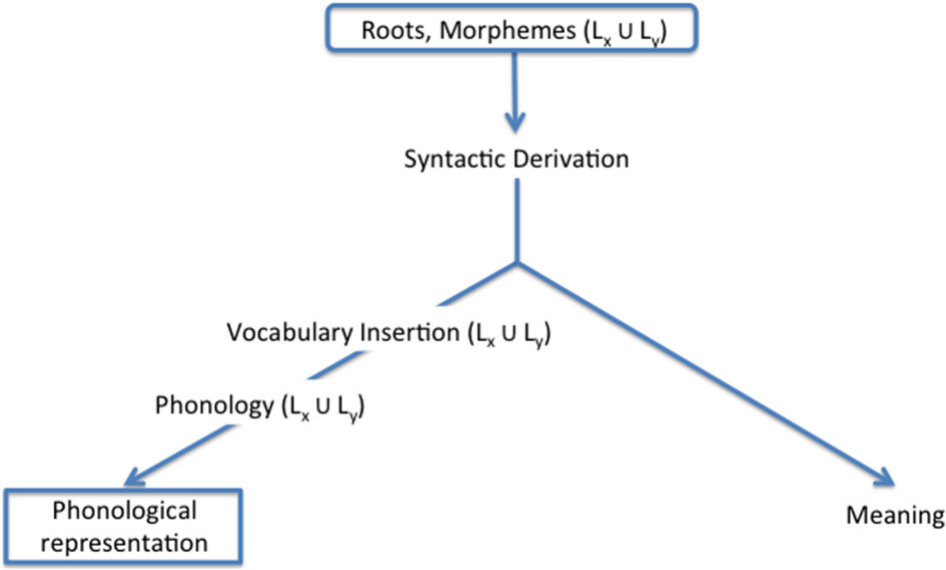
**Synthesis model (Lillo-Martin et al., 2012; Quadros et al., 2013)**.

In a series of works, we have been developing such a model (Lillo-Martin et al., 2010, 2012; Koulidobrova, 2012; Quadros et al., 2013). Our model, illustrated in Figure 1, adopts the viewpoint that bilingualism should be explained using the same architecture of linguistic behavior as required for monolinguals (MacSwan, 2000, 2005). Bilinguals simply have additional materials to work with, but they must adhere to the overall grammatical possibilities and constraints placed on any language. We start with a standard generative perspective incorporating concepts of distributed morphology (Halle and Marantz, 1993; Chomsky, 1995). The input to a derivation contains abstract roots and morphemes. For a bilingual, there are two sets of items to choose from for every derivation. During the syntax, featural requirements must be satisfied; and in some cases, elements from language A may satisfy the requirements of elements from language α, leading to structures with cross-linguistic influence or transfer. At the point of Vocabulary Insertion, elements from either language may be inserted, as long as all featural requirements are satisfied, leading to code-switching. Finally, when two independent sets of articulators are available, lexical items from both languages are possible, making code-blending possible. All three of these outcomes are considered natural consequences of our Synthesis model, so-called because it offers a picture of the combinatorial possibilities allowed by the language architecture.

Our project tests the usefulness of this model in explaining the development of bimodal bilingualism. We have found that hearing children acquiring a sign language and a spoken language (kodas—kids of Deaf adults) engage in the types of productions predicted by the Synthesis model: transfer, code-switching, and code-blending (see references cited in previous paragraph). Given that code-blending is an option available to bimodal bilinguals and not to unimodal bilinguals, we now raise the question whether the process of developing interlocutor sensitivity and discourse separation of languages is different for these two groups of children. How do koda children employ code-blending in their developing language selection? In addition, since parents and other interlocutors vary in their own use of code-blending, how do children adjust to the modality of the input in a given situation?

In this article, we address this question by presenting data from our study on the development of bimodal bilingualism in children learning one of two language pairs: American Sign Language (ASL) and English (Eng) in the US, or Brazilian Sign Language (Libras) and Brazilian Portuguese (BP) in Brazil. The data from two children for each language pair indicate that 2-year-old kodas are sensitive to their interlocutor, and modulate their language choice accordingly, but they are also influenced by the fact that the spoken languages are dominant in their broader community and they do not simply mirror the language choices of their interlocutors. Note that our use of the term “choice” is not meant to necessarily imply a conscious decision; it is simply the term to describe the language used by the child or adult in a particular situation.

## Background

### Previous studies on language choice in the development of unimodal bilinguals

Studies of unimodal bilingual children have found that they typically display interlocutor sensitivity at an early age, using more of language A with an interlocutor who speaks A, and more of language α with an interlocutor who speaks α (Genesee et al., 1995; Petitto et al., 2001). This does not mean that the child will only use A or α with speakers of A or α, respectively, or even mostly A/α in the “appropriate” environment. As Paradis and Nicoladis (2007, p. 278) summarize, “Interlocutor sensitivity, then, is not the same as perfect separation of language by discourse context (discourse separation).”

The child's degree of interlocutor sensitivity changes over the early years. At the earliest ages (before 2;0), children's language choices may be attenuated by their lexical knowledge, since a certain amount of code-switching might take place to fill lexical gaps (Deuchar and Quay, 1999; Nicoladis and Secco, 2000). Deuchar and Quay argued that when lexical knowledge is taken into consideration (i.e., considering whether or not the appropriate language is used when the child knows the word in both languages), “there is a strong tendency for the language of the child's utterances to match that of the context” as early as 1;07–1;08.

Genesee et al. (1995) and Nicoladis and Genesee (1996), and others have observed that 2-year-old bilingual children generally demonstrate interlocutor sensitivity. During this period, there are several factors presumed to contribute to the degree of sensitivity and discourse separation children display. One factor is language dominance: children are more likely to use their dominant language in the contexts calling for it than they are to use their own non-dominant language in its contexts (Genesee et al., 1995; Nicoladis and Genesee, 1996). Another relevant factor is the communication style used in the home. When parents are more tolerant and indicate understanding when their children code-mix or choose the “inappropriate” language (sometimes known as a bilingual strategy), children may display less discourse separation, compared to families who are more strict in their expectations about language choices (that is, they pursue a one-parent one-language or “monolingual” strategy) (Döpke, 1992; Lanza, 1997).

Some studies report a high degree of sensitivity and control over language choice at a relatively early age. Comeau et al. (2003) studied six 2-year-old French-English bilinguals (2;00–2;07; mean 2;05). In their study, an experimenter interacted with the children on three separate occasions, deliberately modifying her rate of code-mixing from 15% of the time in the first session, to 40% in the second session, and back to 15% in the third session. Remarkably, they found that five of the six children matched the changes in proportion of mixing overall, and almost all comparisons showed that the children were more likely to use a mixed utterance following a mixed utterance by the interlocutor, and a non-mixed utterance following a non-mixed utterance. These results demonstrate a very early ability to make language choice selections to match those encouraged by the context.

**Figure 2:**
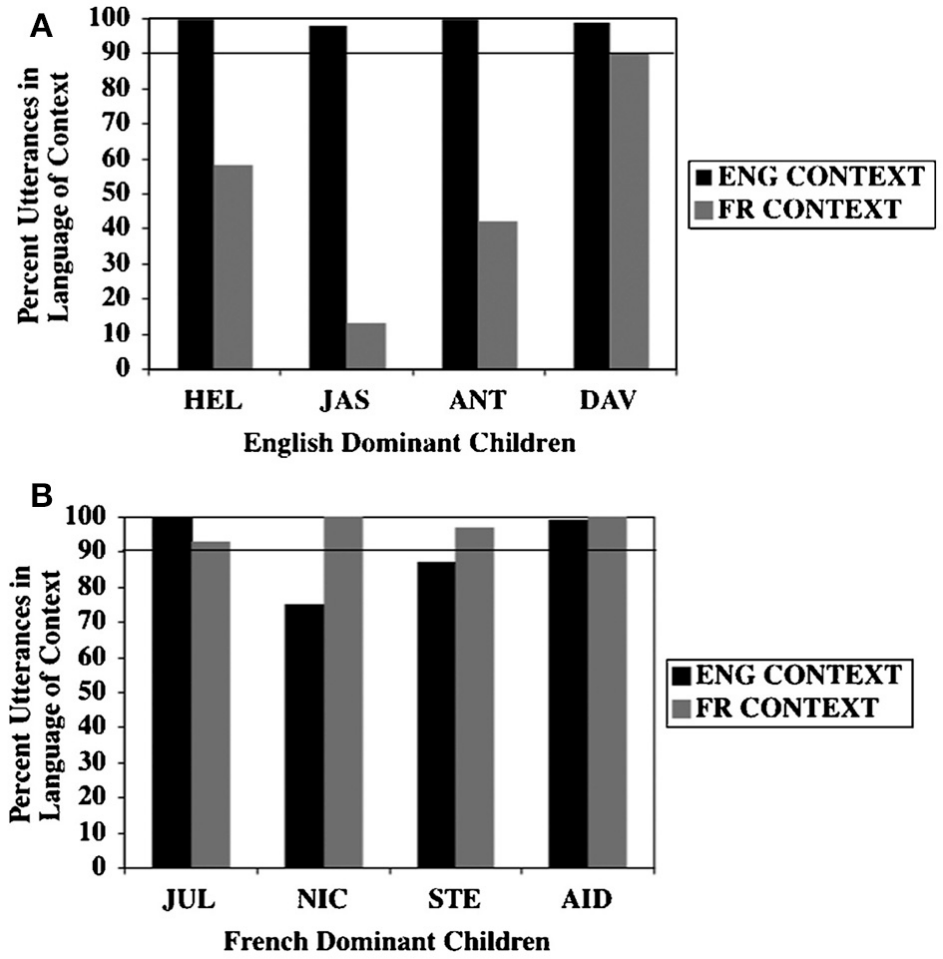
**Unimodal bilinguals**. Percent use of utterances in the language of the context by **(A)** English-dominant children and **(B)** French-dominant children in English and French contexts (Paradis and Nicoladis, 2007) (Reproduced with permission from Taylor and Francis).

One study examined the interlocutor sensitivity of slightly older children, in order to determine whether true discourse separation can be achieved in the preschool years. In addition to taking into consideration children's relative language dominance, this study also considered the factor of community dominance. Paradis and Nicoladis (2007) studied eight children, ages 3;06–4;11, in the English-dominant English-French bilingual community of Alberta, Canada. In this broader context, people are more likely to use English-only with English-speaking interlocutors, with some mixing occurring with French-speaking interlocutors. As expected (see Figure 2), the French-dominant children in this study tended to use French-only in French contexts, and they were highly likely to use English-only in English contexts. On the other hand, while the English-dominant children used English virtually exclusively in the English contexts, they had a lower proportion use of French in the French contexts. Paradis and Nicoladis suggested that the dominance of English in the greater sociolinguistic context contributed to this result; indeed, there was very little mixing in English contexts. In French contexts, more mixing was tolerated, with the children with weaker skills in French responsible for a good deal of this mixing.

### Previous studies on code-blending and language choice in the development of young bimodal bilinguals

Studies of code-blending and language choice for pre-school aged bimodal bilinguals are still fairly rare, although interest in this topic stretches back several decades. All of the previous studies, like ours, focus on kodas—hearing children with at least one Deaf signing parent. Very early investigations included that by Griffith (1985), a longitudinal study of the hearing son of two Deaf American parents, with a Deaf older sibling. Griffith reports that the bimodal bilingual child from the age of 19 months demonstrated “mode-switching” or the use of different language choices according to his interlocutors. Over time, he matched the “mode” most frequently used by each partner, signing more with his sign-dominant father and using sign+speech with his mother, who tended to address him in like manner. Griffith proposed that the child deduced the language preferences of his interlocutors based on whether or not they reacted to his speech only, sign only and sign+speech utterances. Further evidence that the child engaged in such “mode-finding” analysis came from his sessions with new, unfamiliar conversational partners, during which he appeared to try out various conversational modes and watch for the reaction of his interlocutor. Overall, Griffith concluded that her bimodal bilingual subject displayed considerable and early communicative competence in selecting an appropriate communication mode according to his interlocutor(s).

**Figure 3:**
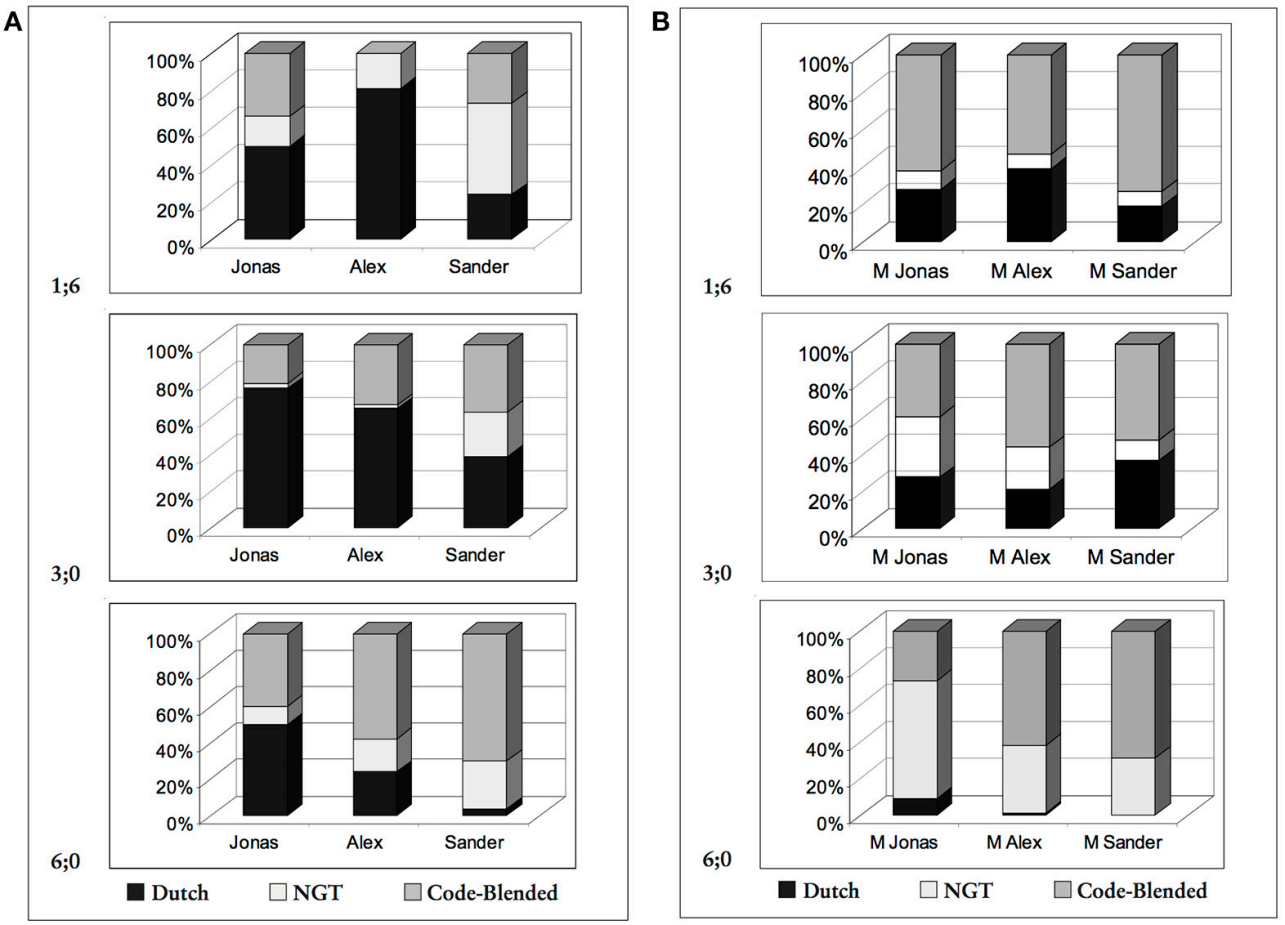
**Bimodal bilinguals**. Language choice by **(A)** hearing children and **(B)** deaf mothers at ages 1;06, 3;00, and 6;00 (van den Bogaerde and Baker, 2009) (Reproduced with permission from Gallaudet University Press).

More recent investigations on code-blending reveal a more complicated picture of developing language choice among very young bimodal bilinguals. In a series of reports on their longitudinal, spontaneous production data from three Dutch hearing children and their Deaf mothers, van den Bogaerde and Baker (2005, 2009; also van den Bogaerde, 2000; Baker and van den Bogaerde, 2008) pointed out that language usage patterns do not necessarily remain static, and that language choices of both bimodal bilingual children and their mothers can change over time (see also Kanto et al., 2013). van den Bogaerde and Baker (2009) reported that the mothers in their study all used a high and fairly consistent percentage of code-blended utterances with their children across three sampling times (when the children were aged 1;06, 3;00, and 6;00). All three mothers also increased their use of NGT-only (Sign Language of the Netherlands) production over time. The bimodal bilingual children in the study increased their use of code-blending overall between 1;06 and 6;00 to levels similar to their mothers', but the same was not true for their production of NGT-only utterances. Two of the three children also continued to produce a much greater proportion of spoken Dutch utterances by 6;00 than was present in their mothers' input. These patterns are illustrated in Figure 3, showing the production of Dutch, NGT and code-blended utterances over time by the children and their mothers, respectively. Note that van den Bogaerde and Baker did not consider phonation to be a criterion for code-blending. Thus, signed utterances accompanied by mouthing of Dutch words, even in the complete absence of any voicing, were counted as code-blending in their data. While some researchers also adopt this practice (e.g., Fung, 2012, studying code-blending in Hong Kong Sign Language and Cantonese), most others (including us) either explicitly or implicitly consider an utterance to include blending only if sign is accompanied by speech with phonation or at least whispering (e.g., Petitto et al., 2001; Emmorey et al., 2008a; Bishop, 2010; Chen Pichler et al., 2010; Donati and Branchini, 2013; Kanto et al., 2013; Petroj et al., 2014).

Van den Bogaerde and Baker concluded from their data that the language choices of the bimodal bilingual children can only be partially explained by input patterns. Other potential influences, such as the children's language proficiency in NGT vs. spoken Dutch, number of Deaf members in the immediate family, and changes in language environment (i.e., entry into school, a Dutch-only environment), also exerted only temporary or inconclusive effects on children's code-blending production. On the other hand, the authors observed that the degree to which mothers tolerated being addressed in speech seemed to have an effect on the children's language choices. Support for this idea comes from the one child in the study, Sander, whose language choice over time most closely resembled that of his mother, whom he addressed almost exclusively in NGT or NGT-Dutch blends by 6;00. Van den Bogaerde and Baker noted that Sander's mother often urged him to sign, even when she could understand his speech perfectly well, prompting the authors to propose mothers' choice of a “more monolingual or bilingual strategy” as the best predictor of bimodal bilingual children's language production patterns. This overall conclusion is similar to that discussed earlier with respect to Döpke (1992) and Lanza (1997) studies of unimodal bilinguals.

A similar conclusion was reached by Kanto et al. (2013), who reported that Finnish kodas whose Deaf parents addressed them primarily in sign showed more development in FinSL (Finnish Sign Language) vocabulary and syntactic complexity from 12 to 36 months than their counterparts who were addressed in mixed sign and speech. The former group's sign exposure was also enhanced by regular weekly/biweekly interactions with Deaf individuals besides their parents, although no information was available on the degree to which these other Deaf individuals mixed sign and speech.

**Figure 4:**
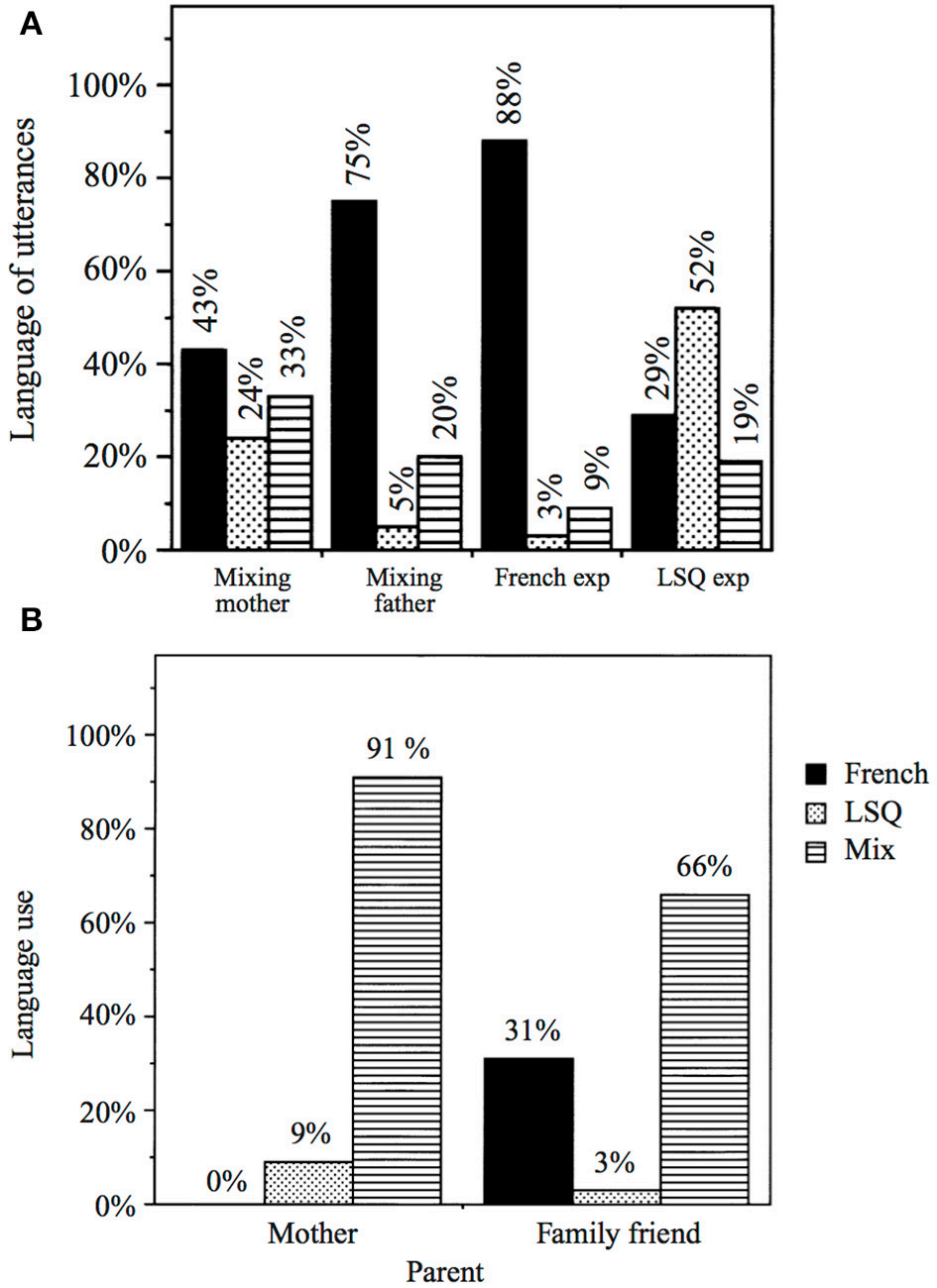
**Bimodal bilinguals**. Language choice by **(A)** one hearing bimodal bilingual child and **(B)** her interlocutors (Petitto et al., 2001) (Reproduced with permission from Cambridge University Press).

Several of the language mixing patterns reported by van den Bogaerde and Baker were also observed by Petitto et al. (2001) for three LSQ (Québec Sign Language)-French bimodal bilingual children and their caretakers. Like the Deaf Dutch mothers, the Deaf caretakers in the Petitto et al. (2001) study also employed a significant degree of code-mixing in their input to their koda children, although the authors did not specify the relative proportions of code-switching vs. code-blending for the parental data. The three children were observed from roughly 0;10–1;08 for the youngest child, 2;10–3;04 for the middle child, and 3;09–4;03 for the oldest child. They were filmed interacting with their Deaf parents, as well as with unfamiliar experimenters who behaved as if they were monolingual in either French or LSQ, allowing observation of the children's reactions to novel communicative environments that called for only spoken language or only sign language. As for their Dutch counterparts, code-blended utterances made up a notable percentage of the utterances these LSQ-French bilinguals addressed to their interlocutors, particularly for the two older children. Petitto et al. (2001) attributed children's degree of mixing directly to the degree of mixing in parental input, citing the relatively high percentages of mixing produced by the second bimodal bilingual subject to her parents (20–33%) and the very high percentage of mixing present in her parents' utterances (66–91%) (see Figure 4 for this child's results). In contrast, French-English comparison bilinguals in their study whose parents addressed them in only one language or the other produced virtually no mixes at all.

However, like Petitto et al. (2001), van den Bogaerde and Baker (2009) concluded that input patterns alone were not sufficient to predict the language choices of their young bimodal bilingual subjects. They cited early sensitivity to interlocutor language and the child's own language preference as two additional factors accounting for the children's language choices. The authors argued that children's sensitivity to interlocutor language could be detected despite the fact that inappropriate language choices were still fairly frequent in the children's production data. Crucially, the children modified their relative proportion use of one language or another across interlocutors with different language needs. This pattern was especially evident in the two data collection conditions in which the children interacted with novel experimenters who behaved as if they were monolingual in either French or LSQ. For instance, Figure 4A shows that although this child used a considerable amount of LSQ and mixing with her parents, she reduced both of these categories dramatically and increased her use of French-only utterances to 88% while interacting with a novel experimenter who spoke only in French. Such modification of proportions of language use was evident from the youngest children in both the LSQ-French and comparison French-English groups. Petitto et al. (2001) argued that the cases of inappropriate language choice were a developmental feature, most likely due to children's language preference and/or temporary lexical gaps, and did not diminish the evidence for “a clear capacity to alter their language choices depending upon the specific language of the addressees, despite differences in degree” (2001, 479).

Petitto et al. (2001) observed that even in blending, children combined signs and speech in semantically appropriate ways to create a cohesive single proposition. Furthermore, when children occasionally produced equivalent strings of signs and speech in different word orders, they chose word orders appropriate for each language. Petitto et al. interpreted such examples as strong evidence that bimodal language mixing was “systematic and principled” (2001, p. 488) from children's earliest utterances, indicating that they differentiated between their two grammars, and refuting popular concerns of language mixing as a sign of language confusion.

Code-blended utterances produced by young kodas are typically quite short, many of them consisting of a single sign plus a single word. In contrast, older children and adult codas are capable of producing much longer code-blended utterances, resulting in more complex interactions between the speech and sign (Emmorey et al., 2008a; Donati and Branchini, 2013). In our on-going work, we investigate the code-blending produced by the children in our project in more detail.

### Expectations for the current study

Taking into consideration the previous studies with unimodal and bimodal bilinguals, the present study was designed to investigate a series of research questions about interlocutor sensitivity, the role of the input, and the unique possibilities for language mixing that emerge in the context of bimodal bilingualism.

In particular, bimodal bilinguals, unlike unimodal bilinguals, have the possibility of three modalities of expression: speech, sign, or bimodal. When addressing various interlocutors, children may take into consideration their ability to understand language addressed to them in each modality. In particular, some Deaf interlocutors have limited access to speech, but this varies greatly from person to person. Some Deaf parents may use speech or blending with their children, or may indicate understanding of spoken or blended utterances addressed to them. Others may insist on sign or blending, which permits the message to be conveyed in sign as well as in speech. Thus, for complete discourse separation, bimodal bilingual children might not be expected to use only sign in sign contexts, but a combination of sign and blending. Furthermore, given the possibility that separation is more complete in the language which is dominant for the community, it might be that as bimodal bilingual children develop, they use more speech-only production in speech contexts, even if a greater variety of choices are made in sign contexts. Note that it is not possible for us to take into consideration children's own language dominance, as there is no independent measure available that is comparable across the sign languages and the spoken languages.

Research Question 1: Do developing bimodal bilingual children show interlocutor sensitivity by selecting language modality at differential rates in speech and sign target sessions? In particular, do they show a greater proportion of spoken language in Speech-target sessions and a greater proportion of sign language in Sign-target sessions? The null hypothesis is that children's language selection does not vary by context (target language). Our expectation is that there will be a difference in language selection across different target language sessions.

Research Question 2: If there is any difference between Speech-target sessions and Sign-target sessions, is this influenced by the dominance of the spoken language in the broader sociolinguistic context? Although the child participants in our study have Deaf families and consistent exposure to sign language, they participate in many activities bringing them into contact with hearing people, including relatives, teachers, neighbors, etc. Our expectation is that children will be closer to achieving discourse separation in the spoken language context, but not necessarily so in the sign context, as in the overall results of the study by Paradis and Nicoladis (2007).

Research Question 3: Do bimodal bilingual children match their language choice to that of their interlocutors? The null hypothesis is that there is no difference between children and their interlocutors. However, we expect children not to simply mirror their interlocutors, but to be influenced by a variety of variables in their language selection.

Research Question 4: Does the pattern of language selection change over time as children develop? We are particularly interested in the possibility that children increase their degree of language separation in the later stages of observation. However, it is possible that a fair amount of mixing will still be observed, since the oldest child in our study was still younger than the youngest child in the study by Paradis and Nicoladis (2007).

Research Question 5: Does the pattern of language selection vary for children in the U.S. compared with children in Brazil? Since our report involves four case studies, two from the U.S. and two from Brazil, we can begin to address the question of possible language-specific or culture-specific differences. However, it would be necessary to study a larger group of children to be able to definitively distinguish language or culture effects from individual differences.

## Methods

### Participants

Participants are four male bimodal bilingual children and their adult interlocutors. The children are included in our long-term project, “Development of Bimodal Bilingualism,” through which they have been involved in data collection with us over a period of years. For all of the children, the home language is a sign language (ASL/Libras); all four receive input in a spoken language (Eng/BP) through other relatives, neighbors, and the community.

- BEN (ASL/English) has two Deaf parents, one Deaf older sibling and one hearing older sibling, one Deaf grandparent and three hearing grandparents. His parents characterize the home environment as predominantly ASL, with some sign+speech blending.
- TOM (ASL/English) has two Deaf parents, and one hearing younger sibling, and no other Deaf family members with whom he has regular contact. His parents characterize the home environment as predominantly ASL, with some sign+speech blending.
- EDU (Libras/BP) has two Deaf parents. His mother signs and uses sign+speech blending. She understands his BP very well. His father only signs.
- IGOR (Libras/BP) has a Deaf father and a hearing mother who is fluent in Libras. They predominantly sign at home. The mother signs and blends sign+speech when the father is present, but when they are by themselves, she speaks with him. His father only signs.

For this article, we have analyzed a subset of the videos collected from each child, focusing on the age range (roughly) 1;06–3;06, as detailed in Table 1. The table provides the age, number of sessions, and number of utterances produced by the children and their interlocutors. In the table, two figures are given for number of utterances: the first figure includes all utterances; the second figure gives the number of utterances included in the analysis, excluding utterances consisting simply of interjections, uninterpretable speech/sign, single points, immediate imitations, etc.

**Table 1:** **Participant information**.

**Child**	**Age range**	**Number speech sessions**	**Number sign sessions**	**Total number utterances produced**			
				**Child, speech sessions**	**Child, sign sessions**	**Interloc., speech sessions**	**Interloc., sign sessions**
BEN (US)	1;04–3;06	8	8	4311 [3078]	2672 [1593]	4577 [3646]	3972 [2899]
TOM (US)	1;05–3;01	4	5	941 [432]	1200 [407]	1106 [712]	2121 [1261]
EDU (BR)	2;00–3;03	4	3	2652 [1866]	1180 [835]	2067 [1617]	899 [803]
IGOR (BR)	2;01–3;07	5	4	2764 [1899]	1741 [1100]	3142 [2918]	1657 [1202]

### Data collection

Participants were video-taped to collect as natural a sample as possible of their ordinary language use. Generally, a target language is established for each session (Sign/Speech), and the target language alternated in weekly sampling sessions. Interlocutors for Sign-target sessions were generally the child's Deaf parent(s), with participation from Deaf (or in some cases, coda) research assistants interacting with the target child and/or behind the camera. Interlocutors for Speech-target sessions were generally a hearing research assistant (RA; all were known signers); in IGOR's case it was his mother. In some cases, a hearing signer research assistant was in the room during target Sign sessions, or a Deaf person (RA or parent) was in the room during Speech target sessions. This person generally stayed behind the camera and did not engage the child. More specifically, one hearing signer was in the room for two of BEN's early Sign target sessions, and two of TOM's early Sign target sessions, and his mother or a Deaf RA was in the room for five of BEN's Speech target sessions. We will explain how we took this into consideration in our analyses below. Our goal was to elicit natural language use and to observe any mixing that occurs; we did not try to enforce language separation (see Chen Pichler et al., 2013 for more detail about our filming methods).

### Data processing

Data processing took place in two steps. Our first step involved transcription of the speech and sign, to build up the corpora on which our analysis depends (Chen Pichler et al., 2010; Quadros et al., 2012, 2014). We subsequently added additional coding for specific research purposes.

#### Transcription

Transcription was done in our research laboratories following the procedures and conventions described in Chen Pichler et al. (2010). To summarize our procedure: The ELAN program (http://tla.mpi.nl/tools/tla-tools/elan/; Crasborn and Sloetjes, 2008) was used for all annotations. Our primary goal is to create an annotated video which can be searched and further annotated for particular research goals. First, hearing assistants transcribed the spoken language used by all participants in the video (the target Child, primary interlocutor Adult1, other Child*n* or Adult*n* interlocutors). Ordinary orthography was used with the addition of special symbols as needed. This initial transcription was checked by another research assistant, and any disagreement was resolved by discussion with at least one additional assistant when needed. Next, (near−)native sign assistants annotated the signing produced by all participants in the video. Glosses (Eng/BP) were used to annotate signs, supplemented by additional conventions shared by all transcribers. Both speech and sign annotations were checked again through additional steps of the process. Utterances were identified as speech and/or sign, with a relatively wide net including all potential linguistic expressions in the initial transcription. Utterance breaks were determined using prosodic information as well as propositional information. Finally, a Free Translation tier was constructed taking into account both the sign language and the spoken language.

#### Coding

For the present analysis, coding required adding the following tiers to our basic ELAN template, with a set for each participant (Child, Adult1, and additional Adult*n* as needed):

- Modality: modality of the utterance (Sign (only), Speech (only), Bimodal, or Excluded)
- Interlocutor: the addressee (Deaf adult, hearing adult, parent, or target child)

Utterances were Excluded if they were completely unintelligible, or consisted of only spoken or signed routines, interjections, non-speech communicative vocalizations or non-sign communicative actions (gestures), or complete imitations of the interlocutor's immediately previous utterance—with no other speech or sign. For example, a spoken “well,” “yes,” “no,” a head nod, an “oops” gesture, or a clap, if occurring by itself, was Excluded. For the Modality analysis, utterances were also Excluded if modality could not be determined; e.g., there was audible speech but the speaker's hands were off-camera.

To count as “bimodal,” we required that some portion of an utterance be presented in sign, and some portion in the spoken language, whether full voice or whispered. It was possible for us to clearly distinguish between full voice and whispering vs. mouthing in the auditory component of our recordings. In whispering, there is turbulence during speech which is not present during mouthing. We did not count mouthing with sign as bimodal (unlike van den Bogaerde, 2000; van den Bogaerde and Baker, 2005, 2009; Baker and van den Bogaerde, 2008). Mouthing with sign in ASL and Libras is quite variable. Mouthing is considered by some to be a mark of influence from the spoken language, and an indication that “contact signing” is being used (cf. Lucas and Valli, 1992). However, many Deaf native signers use mouthing frequently, and more and more linguistic analyses have treated mouthing as a part of the sign language (e.g., Nadolske and Rosenstock, 2007). From our perspective, mouthing may sometimes be a mark of bilingualism, but it is also sometimes a part of signing. Our decision to require full voice or whispering for an utterance to qualify as bimodal obviates the need to judge the status of specific instances of voiceless mouthing. Of course, it means that our figures for proportion of blending cannot be directly compared with those of researchers who include mouthing (e.g., van den Bogaerde and Baker references cited above).

In the initial analysis, combinations of speech and sign interjections (e.g., spoken “yes” with a head nod) were counted as Bimodal, as were combinations of speech with only an index/pointing sign. In subsequent analyses, such combinations were not included in bimodal counts.

For each included utterance, interlocutor was determined by examination of the video. In most cases, the child is addressing the primary interlocutor and vice-versa. Occasionally, a different interlocutor is addressed; for example, the interlocutor might address the cameraperson to check the status of the cordless microphone. In some cases, more than one interlocutor is present, such as when the child is filmed with both Deaf parents.

All of the Brazilian data was coded once by a single coder, and checked and modified by a second coder. Most of the US data was coded by a single coder, with another experienced coder providing coding for a small subset of the data. To check reliability, 5% of the US data were coded blind by a second coder. After the two codings were compared by a third experienced coder, it was determined that accuracy of coding modality was over 93%, and interlocutor coding was over 97% accurate.

## Results

### Overall analysis

For our first analysis, we calculated the proportion of sign, speech, and bimodal utterances produced by the children and their interlocutors across all contexts within speech target sessions and sign target sessions. The results of this calculation are presented in Table 2. Two things are immediately clear. First, the children showed differentiated production in Speech vs. Sign target sessions. This is confirmed by a series of four 2 × 3 chi-square tests of independence (*n* ranged from 943 to 4671, χ^2^ = 163.5–1512.58, *p* < 0.0001 for all four tests, Cramer's *V* = 0.3123–0.5683). Second, the children were distinct from their interlocutors in their patterns of speech, bimodal, and sign production in both Speech and Sign target sessions (for seven of eight chi-square tests, *n* = 1356–6724, χ^2^ = 54.21–1130.18, *p* < 0.0001, Cramer's *V* = 0.1574–0.8128; the effect is marginal at χ^2^_(2, *n* = 4813)_ = 5.82, *p* = 0.0545, Cramer's *V* = 0.0348 for the comparison between IGOR's output and that of his interlocutors in Speech sessions).

**Table 2:** **Overall results**.

		**BEN**		**TOM**		**EDU**		**IGOR**	
		**Child**	**Interlocutor**	**Child**	**Interlocutor**	**Child**	**Interlocutor**	**Child**	**Interlocutor**
Speech target	Speech	0.58	0.73	0.81	0.75	0.93	0.74	0.78	0.75
	Bimod.	0.37	0.24	0.13	0.24	0.06	0.21	0.18	0.20
	Sign	0.05	0.03	0.06	0.00	0.01	0.05	0.04	0.05
Sign target	Speech	0.11	0.05	0.41	0.01	0.65	0.02	0.51	0.03
	Bimod.	0.43	0.10	0.36	0.10	0.17	0.03	0.29	0.08
	Sign	0.45	0.85	0.23	0.89	0.18	0.96	0.20	0.89

*Proportion of speech, bimodal and sign utterances produced by each child and interlocutors in Speech target sessions and Sign target sessions*.

### Developmental analysis

To further investigate the patterns of language mixing by bimodal bilinguals, as distinct from other hearing children, we conducted a second analysis in which we eliminated bimodal utterances where speech was accompanied by pointing, but no other sign. Such speech+pointing combinations are not unique to bimodal bilingual children, as they are commonly reported in studies of hearing, non-signing children (Capirci et al., 1996; Ozçaliskan and Goldin-Meadow, 2005), where points accompanying speech are classified as gesture. Our elimination of speech+point combinations from the second analysis was a conservative measure, given the considerable debate in the sign linguistics field over the status of pointing in sign language. For the same reason, we also excluded combinations consisting solely of elements that would be Excluded if occurring alone (e.g., sign+speech interjections or speech+gesture).

**Figure 5:**
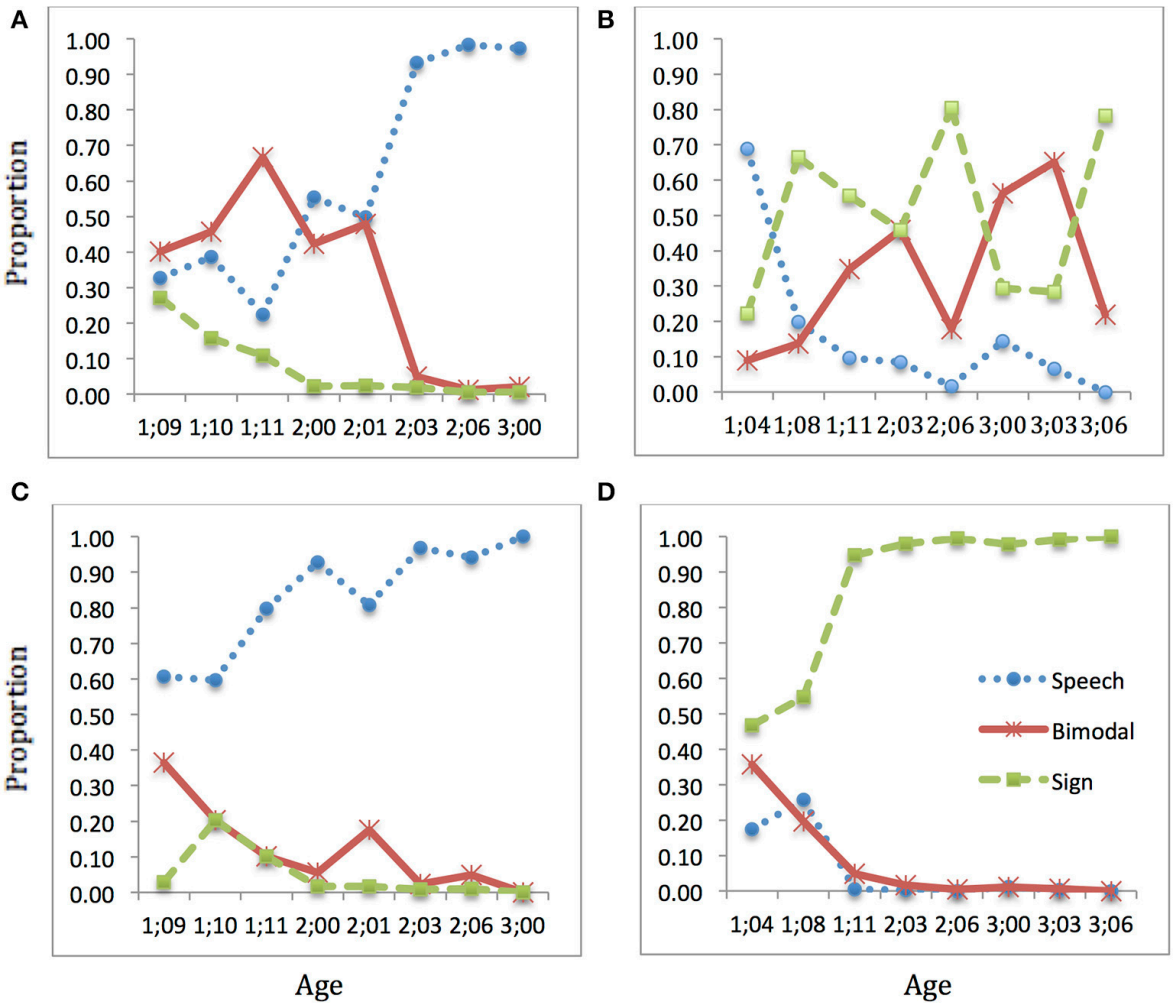
**Language choice over time: BEN**. Proportion of speech (blue dotted), sign (green dashed), and bimodal (red solid) utterances produced: **(A)** child speech target; **(B)** child sign target; **(C)** interlocutor(s) speech target; **(D)** interlocutor(s) sign target (Note: Intervals along x-axis are not regular).

**Figure 6:**
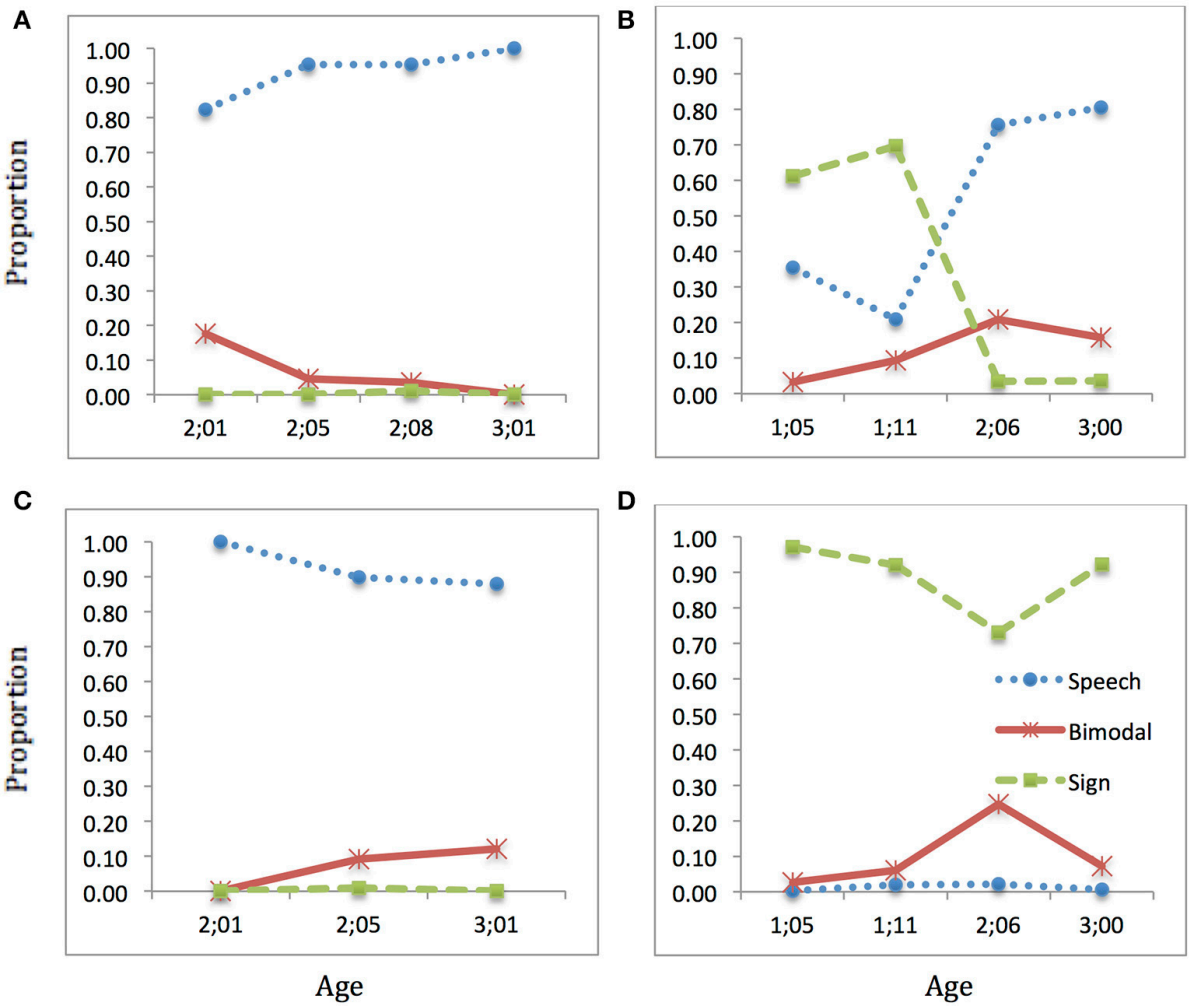
**Language choice over time: TOM**. Proportion of speech (blue dotted), sign (green dashed), and bimodal (red solid) utterances produced: **(A)** child speech target; **(B)** child sign target; **(C)** interlocutor(s) speech target; **(D)** interlocutor(s) sign target (Note: Intervals along x-axis are not regular).

**Figure 7:**
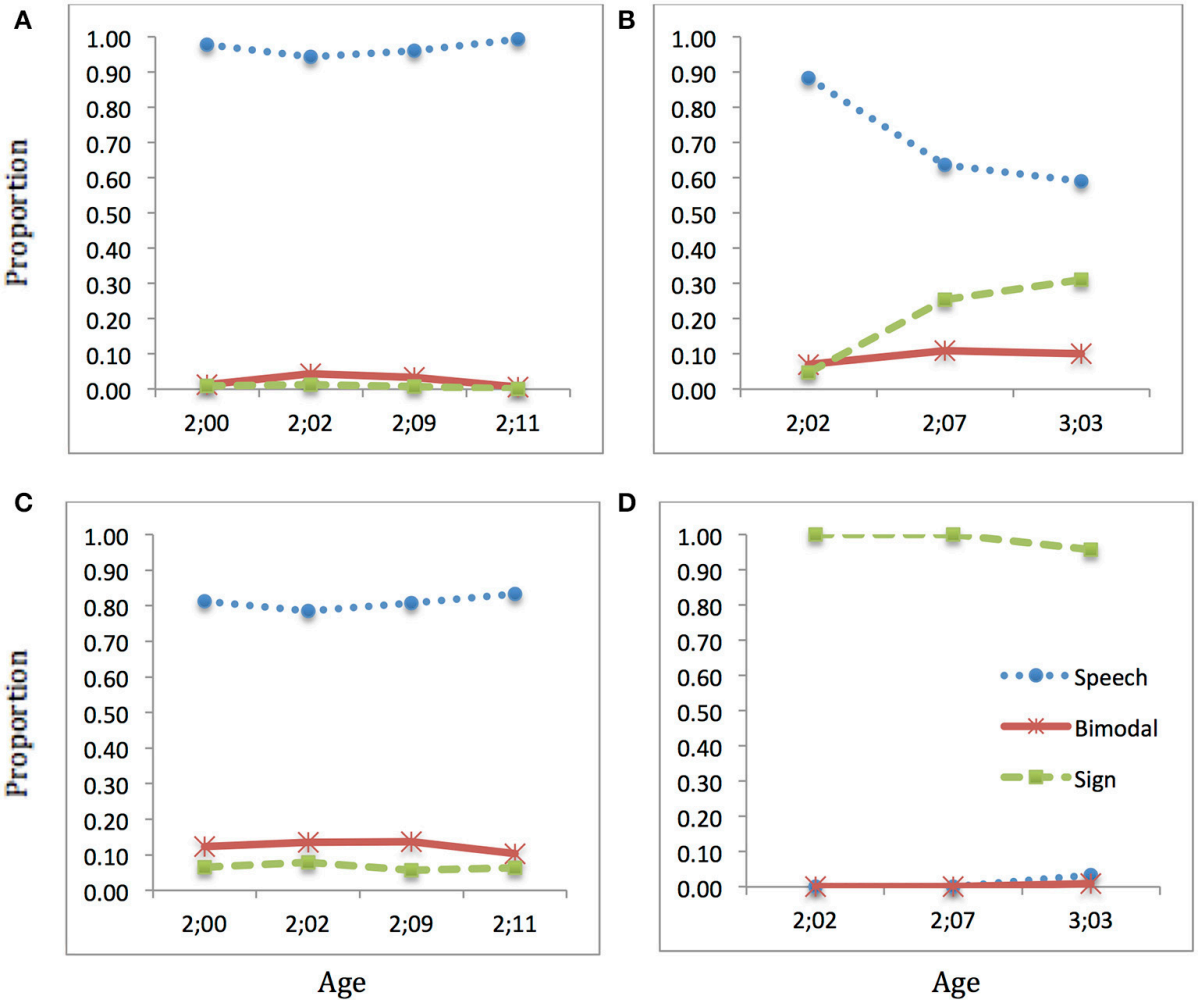
**Language choice over time: EDU**. Proportion of speech (blue dotted), sign (green dashed), and bimodal (red solid) utterances produced: **(A)** child speech target; **(B)** child sign target; **(C)** interlocutor(s) speech target; **(D)** interlocutor(s) sign target (Note: Intervals along x-axis are not regular).

**Figure 8:**
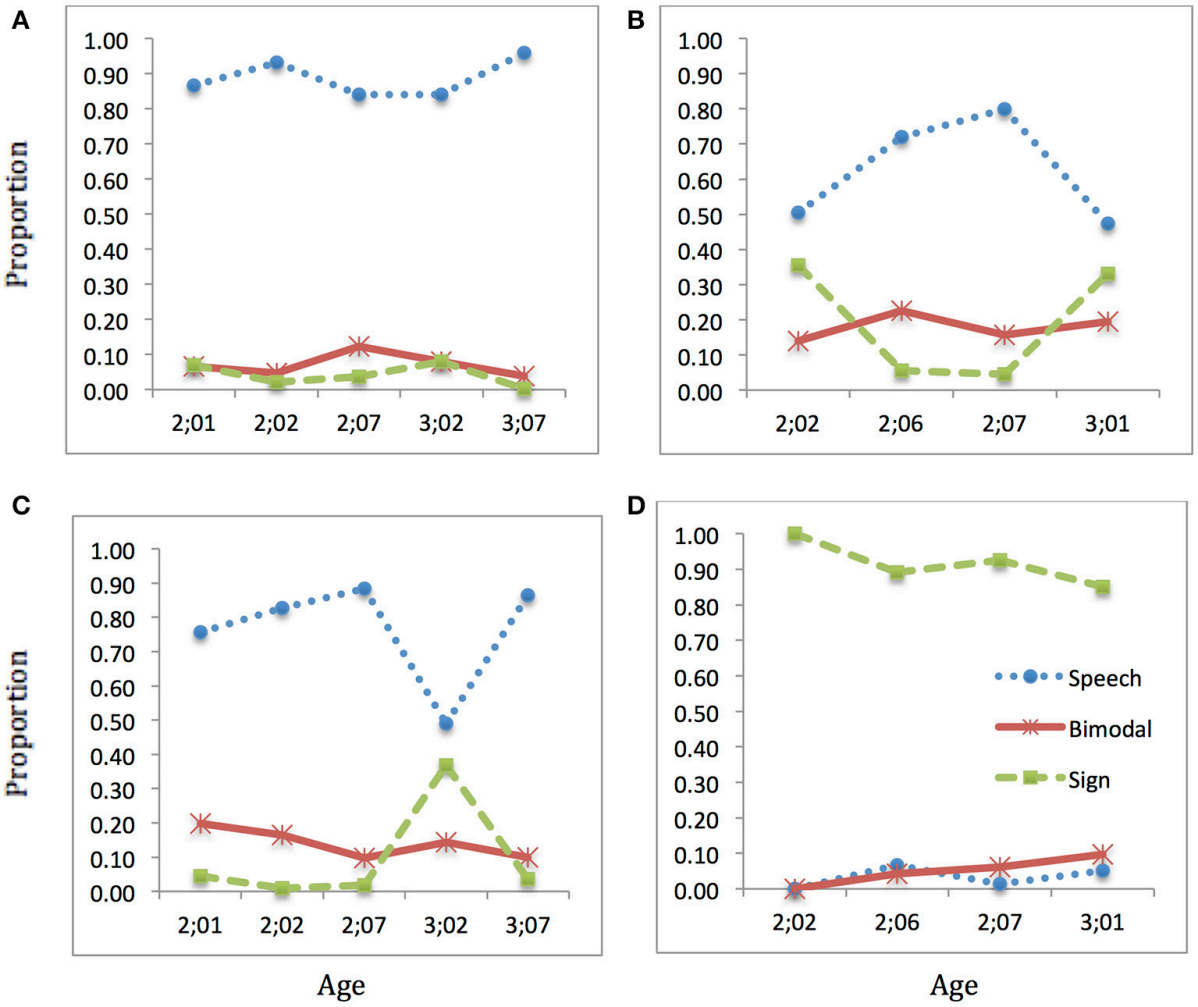
**Language choice over time: IGOR**. Proportion of speech (blue dotted), sign (green dashed), and bimodal (red solid) utterances produced: **(A)** child speech target; **(B)** child sign target; **(C)** interlocutor(s) speech target; **(D)** interlocutor(s) sign target (Note: Intervals along x-axis are not regular).

In addition, we separated out utterances addressed to different interlocutors. In particular, the US sessions occasionally included multiple interlocutors with different auditory status. We focused on the children's productions to hearing interlocutors in the Speech sessions and to Deaf interlocutors in the Sign sessions. We also focused on the interlocutors' utterances to the target child, excluding those addressed to other participants. Finally, we calculated the proportion of speech, bimodal, and sign productions at each session, in order to observe possible developmental effects. The results of these calculations are displayed graphically in Figures 5–**8**.

A series of chi-square tests were applied to see whether the modality pattern (speech, bimodal, sign) produced by each child was different from that produced by the interlocutor(s) in the same session. A second series of chi-square tests examined the difference between each child's own productions in Speech target sessions and Sign target sessions at comparable ages. A full table of the results of these comparisons is available in the Supplementary Materials for this article. The results are summarized in Table 3.

**Table 3:** **Developmental results**.

		**Number comparisons**	**Number comparisons significant at *p* < 0.05**	**Number comparisons significant at *p* < 0.0001**
BEN	Speech vs. input^a^	6	5	5
	Sign vs. input^b^	7	7	6
	Speech vs. sign	4	4	4
TOM	Speech vs. input^b^	2	1	1
	Sign vs. input^a^	2	2	2
	Speech vs. sign^a^	1	1	1
EDU	Speech vs. input	4	4	4
	Sign vs. input	3	3	3
	Speech vs. sign	2	2	2
IGOR	Speech vs. input	5	4	4
	Sign vs. input	4	4	4
	Speech vs. sign	3	3	2

*Comparisons between Child vs. Input for Speech, Child vs. Input for Sign, Child Speech vs. Child Sign*.

a *Two additional comparisons could not be conducted because two or more expected cell frequencies were calculated to be smaller than 5*.

b *One additional comparison could not be conducted because two or more expected cell frequencies were calculated to be smaller than 5*.

To summarize: with very few exceptions, virtually every comparison showed a significant difference between each child and his interlocutors, and between each child's own productions in speech and sign target sessions.

## Discussion

Let us interpret the results of our analyses within the context of the five research questions raised in the Section called Expectations for the Current Study.

Research Question 1: Do developing bimodal bilingual children show interlocutor sensitivity by selecting language modality at differential rates in Speech- and Sign-target sessions? In particular, do they produce a greater proportion of spoken language in Speech-target sessions and a greater proportion of sign language in Sign-target sessions? Our prediction was that, counter to the null hypothesis, children would differ in language selection across different target language sessions.

Our expectation in this case was confirmed by our overall analysis presented in Table 2. The children did indeed differ in their language selection across contexts, with each child producing more speech in the Speech-target sessions than in the Sign-target sessions, and more sign in the Sign-target sessions than in the Speech-target sessions.

Research Question 2: Is any difference between Speech-target sessions and Sign-target sessions influenced by the dominance of the spoken language in the broader sociolinguistic context? Our expectation was that children would be closer to achieving discourse separation in the spoken language context, but not necessarily so in the sign context. This expectation was also borne out. Looking at the results in Table 2 again, we see that each child had a higher proportion of speech in the Speech-target sessions than their proportion of sign in the Sign-target sessions. In the overall analysis, three of the children (TOM, EDU, and IGOR) had over 75% use of speech in Speech sessions, but less than 25% use of sign in Sign target sessions. In this respect, their overall performance was comparable to the degree of language separation exhibited by three of the English-dominant children in the study by Paradis and Nicoladis (2007; cf. Figure 2).

There is a possible alternative explanation for our observation that children were closer to achieving discourse separation in the spoken language context than in the sign context. Rather than a function of the strong dominance of spoken language in the broader sociolinguistic context, this finding could be due to some kind of special tuning of the human linguistic system that preferences speech over sign. The possibility that the human linguistic system preferences speech might be supported by the observation that sign languages are reserved for contexts in which spoken language won't do—Deaf communities, and hearing communities that for various reasons don't speak (e.g., certain religious orders, or persons working in very loud conditions). To the contrary, some researchers have explicitly argued that the human linguistic system is amodal, equipotential for input in a sign language or a spoken language (Petitto and Marentette, 1991).

If the human linguistic system has a preference for spoken language, we might well expect hearing people to uniformly show this preference, despite having input in a sign language from birth. They might even be expected to have difficulty switching from the preferred, dominant language to the less preferred one.

Indeed, Emmorey et al. (2008b) find that their Coda participants in general show dominance in speech, based on self-report of skill level, and psycholinguistic task responses. However, these findings represent the participants in their experiments as a whole. It is not the case that every individual showed the same pattern, and the self-report ratings for proficiency in speech vs. sign are very close. One participant in the study by Emmorey et al. (2008a) responded to one of the tasks using ASL only. In addition, anecdotal reports by adult codas indicate that many consider ASL to be their primary language (Bishop, 2010).

Even if signed and spoken language are equipotential (Petitto, 1994), it would not be surprising to find a strong tendency for hearing native signers to be (or become) speech dominant. Even for those who work in an environment with others using sign language (e.g., a school), a truly balanced or sign-dominant environment would be rare. In the absence of a method to control for or counterbalance such a potentially overwhelming factor, data on the dominance of speech vs. sign in bimodal bilinguals will not be able to rule out (or support) the hypothesis that an overall linguistic preference for speech is at work. Nevertheless, taking into consideration individual differences in the strength of the asymmetry between speech and sign, we will continue to consider the environment as a primary causal factor.

Research Question 3: Do bimodal bilingual children match their language choices to that of their interlocutors? The null hypothesis is that there is no difference between children and their interlocutors, but our overall analysis revealed a significant difference (Table 2). Of the eight comparisons between the four children and their interlocutors in Speech and Sign sessions, seven were highly significant and one (IGOR speech) was marginal. However, visual inspection of the numbers in Table 2 makes it clear that the patterns of usage for the children are much closer to those of their interlocutors in the Speech sessions than in the Sign sessions. In addition, the values for Cramer's V are much higher for the Sign sessions (range 0.4222–0.8128) than for the Speech sessions (range 0.1574–0.2623 for the three significant results), indicating that the differences between the children and their interlocutors are much higher in the Sign sessions.

We take these results as a strong indication that children's language choice is a function of their developing knowledge of the two languages and their appropriate contexts of usage. We will return to this point in the discussion of Research Question 4.

Research Question 4: Does the pattern of language selection change over time as children develop? For this question, we refer to the developmental analysis presented in the Section called Developmental Analysis. As the graphs indicate, the children's choices did change over time, but in different ways for each individual child. We discuss each child's results in turn.

### BEN

The results presented in Figure 5 show that in Speech sessions, BEN's use of sign started relatively low and declined quickly to essentially zero by age 2;00, but his use of bimodal utterances continued along with speech until 2;03, from which point he achieved complete discourse separation for Speech. It is interesting to note that his interlocutors' use of sign and bimodal utterances was also relatively high during the earliest sessions, with sign reaching zero by 2;00 and bimodal by 2;03. In this respect, BEN and his interlocutors were similar, but it is not clear whether it was BEN's use of sign and bimodal productions that encouraged the interlocutors to use these modalities or vice-versa. We note that at 2;03, the statistical comparison did not show a significant difference between BEN and his Speech interlocutors (it was marginal at *p* = 0.063), and at the two later ages, the chi-square test could not be done because of low expected frequencies in two cells—this in turn being due to the low use of sign by both BEN and interlocutors. Thus, he clearly moved toward the same pattern of production in Speech sessions (speech only) as his interlocutors did.

The picture is quite different, and very interesting, in BEN's Sign sessions. First, we observe that BEN's mother (the primary interlocutor in all but the last one of the Sign sessions reported here) made a notable change in her own productions. In fact, she reported to us that she originally thought it would be best to use blending with her hearing child, but she decided when he was 1;11 to stop using speech with him and use sign exclusively. The data from our observation sessions indicate that she adhered to this commitment. BEN's own use of speech in Sign sessions decreased dramatically over the early period, and reached a low baseline by 1;11. However, BEN did not use sign exclusively while not using speech in Sign sessions; rather, he used a mixture of sign and bimodal productions. The proportion of sign and bimodal production fluctuated greatly, with no apparent pattern.

As mentioned earlier, in the US sessions occasionally additional research assistants were present in the room but not interacting directly with the children. In order to see whether the presence of other adults in the room affected children's use of speech vs. sign, we checked carefully to see which sessions had additional participants (e.g., a camera-person) and how this relates to language choice. For BEN's Sign sessions, a hearing (signer) was present in the first three sessions only. All of the later sessions—in particular, those showing great fluctuations in the use of sign vs. bimodal productions—had only Deaf people present in the room. There was greater variability in the Speech sessions, with a Deaf person present in five of the eight sessions throughout the observation period. However, as noted, BEN became quite dominant in using speech only during Speech sessions, apparently despite the occasional presence of a Deaf person in the room.

### TOM

TOM's pattern of results, presented in Figure 6, show that he had a high and increasing tendency to use speech only in Speech sessions throughout the observation period. His hearing interlocutors showed a slight trend in the opposite direction, using more bimodal productions over time. It is this difference that likely led to the overall significant difference between TOM and his interlocutors in Speech sessions, even though the differences were not significant in the two earliest sessions (note that at 2;01, neither TOM nor his interlocutor produced very many utterances that could be included in the second analysis, because there were multiple participants in the session and we had to exclude many utterances). There were only hearing interlocutors and people in the room during the Speech sessions coded.

In Sign sessions, a hearing signer was present at the earliest session, but otherwise only Deaf people were present. TOM's interlocutors predominantly used sign, but there was an increase in bimodal productions by his mother, the primary interlocutor in the session at age 2;06. Our informal observations suggest that TOM's mother did use bimodal productions with him and with other people often, so we do not take this to be a misrepresentation of his input in general. TOM's own productions in Sign sessions displayed an increase over time in speech and bimodal production, with a corresponding decrease in sign. Thus, by 3;00 TOM showed a strong tendency to use speech in both types of sessions, while still distinguishing between the two contexts.

### EDU

EDU's pattern of language selection, shown in Figure 7, showed little change over time in Speech sessions. His use of speech was at ceiling in these sessions, despite the notably lower rate of speech and correspondingly higher rate of bimodal and sign productions by his interlocutors. In Sign sessions, EDU started with a high proportion use of speech, but this was moderated over time, moving toward higher use of Sign but relatively low use of bimodal productions. The interlocutors in his Sign sessions—his Deaf mother and father—used sign almost exclusively on camera, but we observed that his mother used speech/bimodal productions with him and with others at other times. Overall, EDU showed a strong speech bias in the observations presented here.

### IGOR

IGOR's developmental data, shown in Figure 8, revealed a fairly constant, high use of speech in Speech sessions. Like EDU, IGOR used more speech than his interlocutors, who also made use of bimodal productions (with an inexplicable increase in the number of sign productions in one session, at 3;02).

In Sign sessions, IGOR used a mixture of speech, sign, and bimodal productions. He appeared to be increasing the amount of sign and correspondingly decreasing the amount of speech by the end of the observation period (3;01). His interlocutors used a high proportion of sign productions, with some bimodal production as well.

Although the details of his production were different, IGOR appears overall to be similar to EDU in showing a strong preference for speech, with movement toward more use of sign and bimodal production in Sign sessions after age 3.

Research Question 5: Does the pattern of language selection vary for children in the U.S. compared with children in Brazil? Since our report involves only four case studies, it is difficult to definitively distinguish language or culture effects from individual differences. Overall, our impression was that TOM, EDU and IGOR showed similarities in performance, as children who favor spoken language and therefore display discourse separation most clearly for their spoken language, but also interlocutor sensitivity for their sign language. Only BEN showed evidence of complete discourse separation for both languages, but this is likely to be an individual difference. No clear language/culture effects were thus observed in our data.

## Conclusions

According to the model of bimodal bilingualism we presented in Figure 1, bilinguals have the option of using grammatical knowledge and lexical items from either language, separately or in combination, as long as general constraints on language structure are met. Further constraints on the use of code-mixing (including code-blending) may be imposed by the sociolinguistic environment: some communities take more advantage of the mixing available to bilinguals, while others tend to avoid it. Thus, children must learn to take into consideration both the structural properties afforded by their languages and the language usage patterns exhibited by individual interlocutors and language communities.

The children in our study showed that they are sensitive to the language used by interlocutors, in that they displayed differential language selection in Speech– vs. Sign-target sessions. Three of the four participants were also strongly affected by the dominance of the spoken language in the broader sociolinguistic community: they distinguished between Speech and Sign contexts, yet showed a preference for use of speech in both contexts. The fourth participant, BEN, showed a full discourse separation pattern, if we count his use of bimodal productions as “appropriate” for the Sign sessions.

One might ask why BEN would use bimodal productions rather than exclusively using sign in Sign-target sessions, given his apparent facility and recognition of the role of sign. Emmorey et al. (2008a) and Pyers and Emmorey (2008), observing adult codas, proposed that codas use code-blending, and even use aspects of ASL non-manual marking while speaking English to non-signers, because (complete) inhibition or suppression of the unselected language has a processing cost. For unimodal bilinguals, use of one language necessitates inhibition of the other language, whereas bimodal bilinguals can use blending to ease the burden of inhibition to varying extents. We suggest that this tendency to blend when inhibition is difficult lies behind BEN's use of blending in the Sign-target sessions. The same might be true for the other three participants, but their rates of blending were overall lower than BEN's.

While the children all showed interlocutor sensitivity, they did not mirror their interlocutors' rates of production of speech, sign, and bimodal utterances. Still, it is quite possible that the attitude of the children's input providers played a role in their language selection, as suggested by Döpke (1992) and Lanza (1997) for unimodal bilinguals, and by van den Bogaerde and Baker (2009) for NGT-Dutch bimodal bilinguals and Kanto et al. (2013) for FinSL-Finnish bimodal bilinguals. In general, the children in our study are exposed to blending from at least one parent, with relatively less sign-only input, and all of the Deaf parents are bilingual to some degree, whether or not they use speech with their hearing child. Many of them also show their understanding of their children's spoken output: for example, EDU's mother answers (in sign) his spoken questions, showing that he can achieve successful communication with her even when he uses speech. In addition, during our data collection sessions the children interacted with numerous hearing people who are known signers, and they also modeled the use of blending. The only case we know where a stricter monolingual strategy is pursued is BEN's mother.

Additional research would be needed to confirm this, but our overall findings are in agreement with those researchers suggesting that greater discourse separation is related to greater adherence to a monolingual strategy. In addition, as Chen Pichler et al. (2014) discuss, maintenance of a minority home language for kodas may be supported through increased opportunities for them to use that language with a variety of interlocutors, including peers, throughout development.

### Conflict of interest statement

The authors declare that the research was conducted in the absence of any commercial or financial relationships that could be construed as a potential conflict of interest.
