# Supplementary material for: Language choice in bimodal bilingual development
Source: Front Psychol. 2014 Oct 20;5:1163. doi: 10.3389/fpsyg.2014.01163 (PMC4202712; doi:10.3389/fpsyg.2014.01163)
Supplement: Supplementary file 1 [file Table1.PDF]

BEN vs. input

Speech Sessions	Age	# Obs	X^2	Cramer's V	p
BEN_017	1;09	752	111.01	0.3842	<.0001
BEN_020	1;10	432	30.56	0.266	<.0001
BEN_021	1;11	911	337.97	0.6091	<.0001
BEN_025	2;00	662	120.22	0.4261	<.0001
BEN_029	2;01	716	76.11	0.326	<.0001
BEN_039	2;03	773	5.53	0.0846	0.063
BEN_048	2;06	1050	--	--	--
BEN_074	3;00	1050	--	--	--
Speech overall		6346	193.88	0.1748	<.0001

Sign Sessions	Age	# Obs	X^2	Cramer's V	p
BEN_002	1;04	247	82.26	0.4948	<.0001
BEN_015	1;08	606	6.98	0.1073	0.0305
BEN_022	1;11	343	92.43	0.4801	<.0001
BEN_041	2;03	467	181.86	0.624	<.0001
BEN_051	2;06	629	--	--	--
BEN_075	3;00	771	410.71	0.7299	<.0001
BEN_091	3;03	642	362.08	0.751	<.0001
BEN_098	3;06	147	21.86	0.3856	<.0001
Sign overall		3852	731.68	0.4255	<.0001

BEN speech vs. sign

Sign Sessions	Speech Sessions	Age	# Obs	X^2	Cramer's V	p
2,15,22	17,20,21	<2;00	980	181.39	0.419	<.0001
41	25,29,39	2;00-2;03	1150	392.33	0.5841	<.0001
51	48	2;06	840	810.46	0.9597	<.0001
75,91,98	74	3;00+	1169	879.36	0.8673	<.0001
	BEN overall		4232	1558.94	0.6069	<.0001
	Input overall		6155	4362.72	0.8419	<.0001

TOM vs. input

Speech Sessions	Age	# Obs	X^2	Cramer's V	p
TOM_028	2;01	23	--	--	--
TOM_039	2;05	813	4.33	0.073	0.1147
TOM_064	3;01	297	22.93	0.2779	<.0001
Speech overall		1219	--	--	--

Sign Sessions	Age	# Obs	X^2	Cramer's V	p
TOM_003+004	1;05	480	--	--	--
TOM_023	1;11	93	--	--	--
TOM_047	2;06	312	205.77	0.8121	<.0001
TOM_061	3;00	446	362.66	0.9017	<.0001
Sign overall		1331	744.42	0.7479	<.0001

TOM speech vs. sign

Sign Sessions	Speech Sessions	Age	# Obs	X^2	Cramer's V	p
23	28	~2	60	24.58	0.6401	<.0001
47	39	~2;06	217	--	--	--
61	64	~3	320	--	--	--
	TOM overall		745	146.9	0.4441	<.0001
	Input overall		1805	1575.2	0.9342	<.0001

EDU vs. input

Speech Sessions	Age	# Obs	X^2	Cramer's V	p
EDU_021	2;00	786	60.18	0.2767	<.0001
EDU_023	2;02	958	54.84	0.2393	<.0001
EDU_033	2;09	798	47.52	0.244	<.0001
EDU_039	2;11	941	80.8	0.293	<.0001
Speech overall		3483	233.39	0.2589	<.0001

Sign Sessions	Age	# Obs	X^2	Cramer's V	p
EDU_024	2;02	603	555.52	0.9598	<.0001
EDU_030	2;07	484	216.88	0.6694	<.0001
EDU_043	3;03	551	260.63	0.6878	<.0001
Sign overall		1638	1018.15	0.7884	<.0001

EDU speech vs. sign

Sign Sessions	Speech Sessions	Age	# Obs	X^2	Cramer's V	p
24	21,23	2-2;02	1200	22.95	0.1383	<.0001
30,43	33,39	2;07-3;03	1501	350.85	0.4835	<.0001
	EDU overall		2701	439.61	0.4034	<.0001
	Input overall		2420	1932.5	0.8936	<.0001

IGOR vs. input

Speech Sessions	Age	# Obs	X^2	Cramer's V	p
IGOR_001	2;01	1044	34.05	0.1806	<.0001
IGOR_004	2;02	1152	30.62	0.163	<.0001
IGOR_025	2;07	1079	5.23	0.0696	0.0732
IGOR_045	3;02	649	98.59	0.3898	<.0001
IGOR_063	3;07	893	27.01	0.1739	<.0001
Speech overall		4817	72.66	0.1228	<.0001

Sign Sessions	Age	# Obs	X^2	Cramer's V	p
IGOR_005	2;02	751	350.6	0.6833	<.0001
IGOR_021	2;06	603	426.31	0.8408	<.0001
IGOR_024	2;07	391	312.43	0.8939	<.0001
IGOR_044	3;01	557	171.21	0.5544	<.0001
Sign overall		2302	1226.92	0.7301	<.0001

IGOR speech vs. sign

Sign Sessions	Speech Sessions	Age	# Obs	X^2	Cramer's V	p
5	1,4	2;0	1101	224.95	0.452	<.0001
21,24	25	2;6	925	12.01	0.1139	0.0025
44	45,63	3;0+	973	200.79	0.4543	<.0001
	IGOR overall		2999	320.22	0.3268	<.0001
	Input overall		4120	2960.2	0.8476	<.0001
